# Association of *PTX3* gene polymorphisms and PTX3 plasma levels with leprosy susceptibility

**DOI:** 10.1186/s12879-023-08862-0

**Published:** 2023-12-05

**Authors:** Ana Clara Cadidé Gonzaga Moraes, Renata Clesia Feitosa Viana da Luz, André Luís Magalhães Fernandes, Milena Xavier Silva Barbosa, Lorena Viana de Andrade, Anderson da Costa Armstrong, Carlos Dornels Freire de Souza, Rodrigo Feliciano do Carmo

**Affiliations:** 1 Postgraduate Program in Health and Biological Sciences, Federal University of the São Francisco Valley (UNIVASF), Pernambuco, Brazil; 2 Foundation of Hematology and Hemotherapy of Bahia (HEMOBA), Bahia, Brazil; 3 Postgraduate Program in Biosciences, Federal University of the São Francisco Valley (UNIVASF), Pernambuco, Brazil; 4 College of Medicine, Federal University of the São Francisco Valley (UNIVASF), Av. José de Sá Maniçoba, s/n, Centro, Petrolina, PE Brazil

**Keywords:** Biomarkers, Leprosy, Pentraxin, Polymorphism, SNP

## Abstract

**Background:**

Pentraxin 3 (PTX3) is a soluble pattern recognition receptor that plays a crucial role in modulating the inflammatory response and activating the complement system. Additionally, plasma PTX3 has emerged as a potential biomarker for various infectious diseases. The aim of this study was to evaluate the association of *PTX3* gene polymorphisms and PTX3 plasma levels with susceptibility to leprosy and clinical characteristics.

**Methods:**

Patients with leprosy from a hyperendemic area in the Northeast Region of Brazil were included. Healthy household contacts and healthy blood donors from the same geographical area were recruited as a control group. The rs1840680 and rs2305619 polymorphisms of PTX3 were determined by real-time PCR. Plasma levels of PTX3 were determined by ELISA.

**Results:**

A total of 512 individuals were included. Of these, 273 were patients diagnosed with leprosy; 53 were household contacts, and 186 were healthy blood donors. No association was observed between *PTX3* polymorphisms and susceptibility to leprosy or development of leprosy reaction or physical disability. On the other hand, plasma levels of PTX3 were significantly higher in patients with leprosy when compared to household contacts (*p* = 0.003) or blood donors (*p* = 0.04). It was also observed that PTX3 levels drop significantly after multidrug therapy (*p* < 0.0001).

**Conclusions:**

Our results suggest that PTX3 may play an important role in the pathogenesis of leprosy and point to the potential use of this molecule as an infection marker.

**Supplementary Information:**

The online version contains supplementary material available at 10.1186/s12879-023-08862-0.

## Background

Pentraxins are evolutionarily conserved proteins that are part of a class of pattern recognition molecules belonging to innate immunity. Pentraxins are classified into short and long pentraxins, with C-reactive protein (CRP) and serum amyloid P component belonging to the short pentraxin group and produced under the stimulus of interleukin (IL)-6 by hepatocytes. Pentraxin-3 (PTX3), on the other hand, belongs to the long pentraxin family, which are rapidly produced in response to pro-inflammatory signals (e.g., TNF-α, IL-1 β), TLR agonists, and microbial recognition [1, 2]. *PTX3* is expressed by various cell types, including myeloid (dendritic cells, monocytes, macrophages, neutrophils), epithelial, vascular and lymphatic endothelial, and mesenchymal (fibroblasts and adipocytes) [2–5].

PTX3 has the ability to modulate the innate immune response involved in protection against infectious diseases, and it acts in the regulation of complement activation, opsonization of pathogens and apoptotic cells [6]. In leprosy, components of the complement system deposited on the surface of *M. leprae* are recognized by CR1 and CR3 receptors and subsequently phagocytized by human monocytes [7]. In addition, serum levels of terminal complement components have been associated with the occurrence of leprosy reaction in Bangladeshi patients [8]. Accordingly, a Brazilian study demonstrated that serum PTX3 levels were higher in multibacillary patients before the development of erythema nodosum leprosum and that thalidomide treatment reduced PTX3 levels [9].

The human *PTX3* gene is located on chromosome 3q25 and is organized into three exons encoding the leader signal peptide, the long N-terminal domain (amino acids 18–178), and the C-terminal pentraxin domain (amino acids 179–381) [1]. Two polymorphisms in the *PTX3* gene (rs1840680 and rs2305619), located in intronic regions and possibly linked to other SNPs in regulatory regions of the gene, have been associated with changes in PTX3 levels and susceptibility to infectious diseases such as pulmonary tuberculosis [10], *Pseudomonas aeruginosa* in Caucasian patients with cystic fibrosis [11], *Aspergillus fumigatus* in patients undergoing hematopoietic stem cell transplantation [12], fungal infections in solid organ transplant patients [13], urinary tract infections [14], and more recently with the severity of COVID-19 [15].

In the present study, we investigated for the first time the association of rs1840680 and rs2305619 polymorphisms in *PTX3* and PTX3 plasma levels with leprosy susceptibility and clinical characteristics.

## Methods

### Study design

This is a retrospective cross-sectional analytical study, conducted in two reference centers in the Northeast Region of Brazil (Dr. Altino Lemos Health Center, in the city of Juazeiro, state of Bahia, and at the Petrolina Infectious Diseases Service, state of Pernambuco), during the period from July 2019 to August 2022. These are hyperendemic cities for leprosy, with an incidence of 22.6 cases per 100,000 inhabitants and 26.6 cases per 100,000 inhabitants in 2013 for Petrolina and Juazeiro, respectively [16]. The study received authorization from the ethics and research committee of the Hospital da Clínicas of the Federal University of Pernambuco (HC/UFPE) under CAAE 66179617.7.0000.5196 and was conducted in accordance with the provisions of the Declaration of Helsinki and Good Clinical Practice guidelines. Written informed consent was obtained from all participants.

All patients were routinely diagnosed according to the Ridley and Jopling criteria. Also, we adopted the World Health Organization classification for treatment purposes, and patients were classified as paucibacillary and multibacillary. Patients were also grouped according to the type of reactive reaction they presented: type I (reversal reaction) and type II (erythema nodosum leprosum). The degree of physical disability was classified as grade 0, grade 1, and grade 2. As a control group, household contacts of patients with no clinical manifestation of leprosy were recruited, as well as healthy blood donors from the same geographical area. Household contact means any individual, related or not, who lives in the same household as the leprosy case. Blood donors with positive serology for hepatitis B or C, syphilis, HIV/AIDS, HTLV, and Chagas disease and household contacts with clinical alterations suggestive of leprosy were excluded. Patients enrolled in the study had no signs or symptoms of tuberculosis.

### Sample collection and processing

A total of 4 mL of blood was collected by venipuncture through a vacuum device into a tube containing EDTA to obtain whole blood and plasma. The collected blood was taken in a thermal box the same day to the Multiuser Research Laboratory (LAMUPE) of the University Hospital of the Federal University of São Francisco Valley (HU-UNIVASF/ EBSERH). In the laboratory, 1 mL of whole blood was separated into 1.5 mL microtubes for the genetic study. The remainder was then centrifuged at 3500 rpm for 10 min to obtain plasma. Immediately after processing, the samples were labeled and stored in a freezer at − 80 °C.

### Genetic study

Genomic DNA was extracted from 200 μl peripheral blood samples using a commercially available extraction kit (ReliaPrep™ Blood gDNA Miniprep System, Promega, Madison, WI, USA), following instructions provided by the manufacturer. The genetic material obtained was stored in a freezer at − 20ºC. The extraction yield and quality of the extracted material was determined using a nanospectrophotometer (Thermo Fisher Scientific). For polymorphism detection, real-time PCR methodology was used using a TaqMan® (Thermo Fisher Scientific) pre-designed probe system for the SNPs rs2305619 and rs1840680 of *PTX3*. The reactions were performed on a QuantStudio™ 5 qPCR instrument (Thermo Fisher Scientific).

### Determination of PTX3 plasma levels

PTX3 concentration was determined in plasma samples using commercial ELISA kits following manufacturer’s instructions (R&D Systems, Quantikine™ Human Pentraxin 3/TSG-14 Immunoassay). A microplate reader (Multiskan™ FC microplate Phometer, Thermo Fisher Scientific) and an automatic microplate washer (Multiwash+, Molecular Devices, Sunnyvale, CA, USA) were used to measure PTX3 levels.

### Statistical analysis

Statistical analysis and graph drawing was performed with GraphPad Prism software version 8.0 (GraphPad Software). Categorical data were expressed as absolute frequency and percentage. Continuous variables were presented as mean and standard deviation. The statistical significance level adopted was *p* < 0.05. The Kolmogorov-Smirnov test was used to verify the normal distribution of continuous variables. Categorical data were analyzed by χ^2^ test or Fisher’s exact test, while comparing quantitative variables between more than two groups, the Kruskal-Wallis test with correction for multiple comparisons (Dunn’s test) was used. For comparison between two groups, the Mann-Whitney U-test was used.

## Results

A total of 512 individuals were included in the present study. Of these, 273 were patients diagnosed with leprosy; 53 were household contacts, and 186 were healthy blood donors. The clinical and demographic characteristics of the three groups are described in Table 1.

**Table 1:** Demographic and clinical characteristics of the investigated groups.

Variable	Leprosy patients (n = 273)	Household contacts (n = 53)	Blood donors (n = 186)
Age (years) ± SD	51.5 ± 14.2	43.8 ± 14.4	35.2 ± 11.2
Sex (male)	160 (58.6%)	14 (26.4%)	105 (56.4%)
Operational classification (n = 270)			
PB	21 (7.7%)	-	-
MB	249 (92.2%)	-	-
Reaction (n = 130)			
No reaction	29 (22.4%)	-	-
Type 1	56 (43.0%)	-	-
Type 2	45 (34.6%)	-	-
Disability grade (n = 178)			
0	81 (45.5%)	-	-
1	52 (29.2%)	-	-
2	45 (25.2%)	-	-

MB: multibacillary; PB: paucibacillary

The mean ages were 51, 43, and 35 years, and the frequency of males was 58.6%, 26.4%, and 56.4% in the groups of patients, contacts, and blood donors, respectively. Among leprosy patients, the multibacillary operational classification was most prevalent 249 (92.2%). A total of 56 (43.0%) and 45 (34.6%) presented type 1 and type 2 reactions, respectively. Regarding the degree of disability, 52 (29.2%) and 45 (25.3%) participants presented grade 1 or 2, respectively (Table 1).

The allelic and genotypic distribution of *PTX3* rs1840680 and rs2305619 polymorphisms among cases, contacts, and controls are presented in Table 2. The polymorphisms studied were in agreement with Hardy-Weinberg equilibrium. There was no significant difference in allelic and genotypic distribution between the groups analyzed (*p* = ns for all comparisons) (Table 2). Also, no significant association was observed between *PTX3* polymorphisms and the occurrence of leprosy reaction or the development of physical disability (data not shown). The median PTX3 levels for the GG, AG, and AA genotypes were 0.88 ng/mL, 0.85 ng/mL, and 0.77 ng/mL for rs1840680 (*p* = ns), and 0.90 ng/mL, 0.84 ng/mL, and 0.79 ng/mL for rs2305619 (*p* = ns) (Supplementary Figure S1).

**Table 2:** Genotype and allele distribution of *PTX3* rs1840680 and rs2305619 polymorphisms among the investigated groups

SNP	Allele/Genotype	Leprosy PB (n = 21/42^1^)	Leprosy MB (n = 249/498^1^)	Leprosy patients (n = 273/546^1^)	Household contacts (n = 53/106^1^)	Blood donors (n = 186/372^1^)	*P* value^2^	*P* value^3^
rs1840680	G	28 (66.6%)	316 (63.4%)	346 (63.3%)	61 (57.5%)	239 (64.2%)	0.74	0.44
	A	14 (33.4%)	182 (36.6%)	200 (36.7%)	45 (42.5%)	133 (35.8%)		
	GG	10 (47.6%)	99 (39.7%)	109 (39.9%)	16 (30.2%)	81 (43.5%)		
	AG	8 (38.1%)	118 (47.3%)	128 (46.9%)	29 (54.7%)	77 (41.4%)	0.70	0.41
	AA	3 (14.3%)	32 (13.0%)	36 (13.2%)	8 (15.1%)	28 (15.1%)		
rs2305619	G	23 (54.7%)	264 (53.0%)	289 (52.9%)	57 (53.7%)	200 (53.7%)	0.92	0.96
	A	19 (45.3%)	234 (47.0%)	257 (47.1%)	49 (46.3%)	172 (46.3%)		
	GG	6 (28.5%)	71 (28.5%)	77 (28.3%)	14 (26.4%)	52 (28.0%)		
	AG	11 (52.3%)	122 (48.9%)	135 (49.5%)	29 (54.7%)	96 (51.6%)	0.87	0.95
	AA	4 (19.2%)	56 (22.6%)	61 (22.2%)	10 (18.9%)	38 (20.4%)		

^1^Allele count; ^2^Leprosy PB vs. Leprosy MB; ^3^Leprosy patients vs. Household contacts vs. Blood donors

PB: Paucibacillary; MB: Multibacillary

**Fig. 1:**
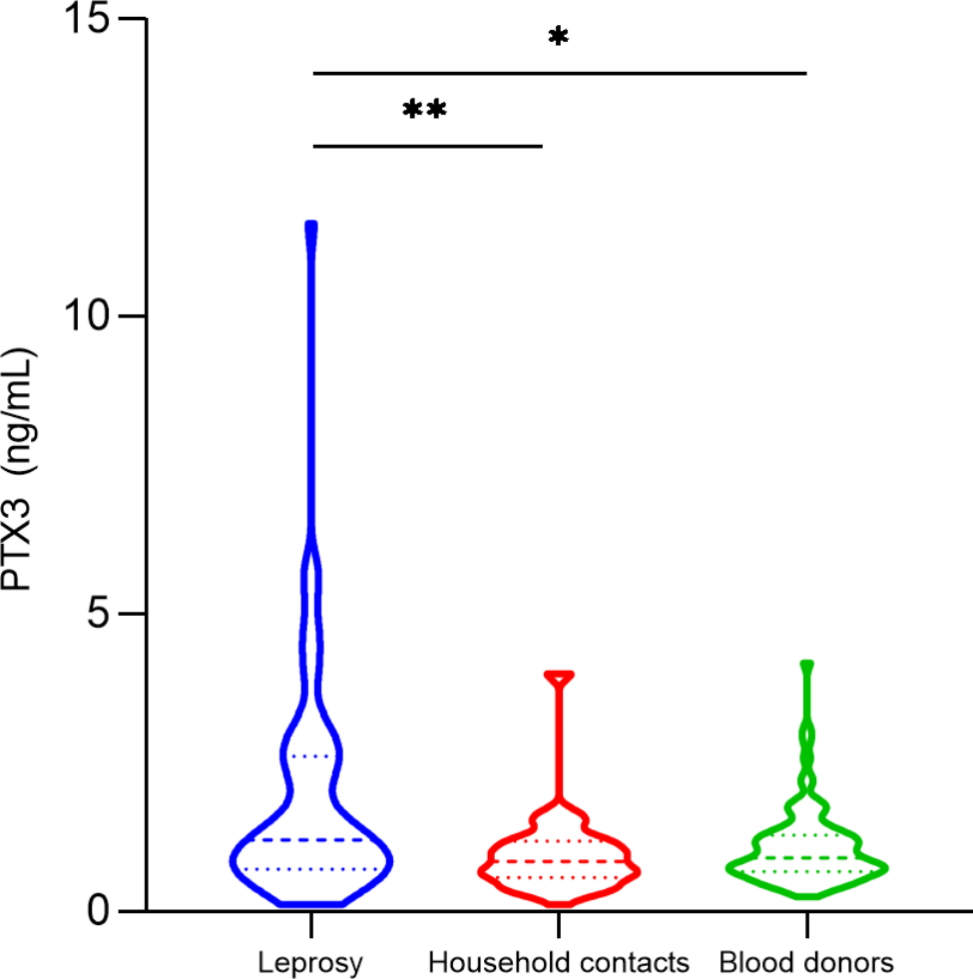
Comparison of PTX3 plasma levels (ng/mL) according to group: patients with leprosy, household contacts, and blood donors. Statistical significance was determined using the Kruskal-Wallis test with post hoc Dunn’s test. *P < 0.05, **P < 0.01

**Fig. 2:**
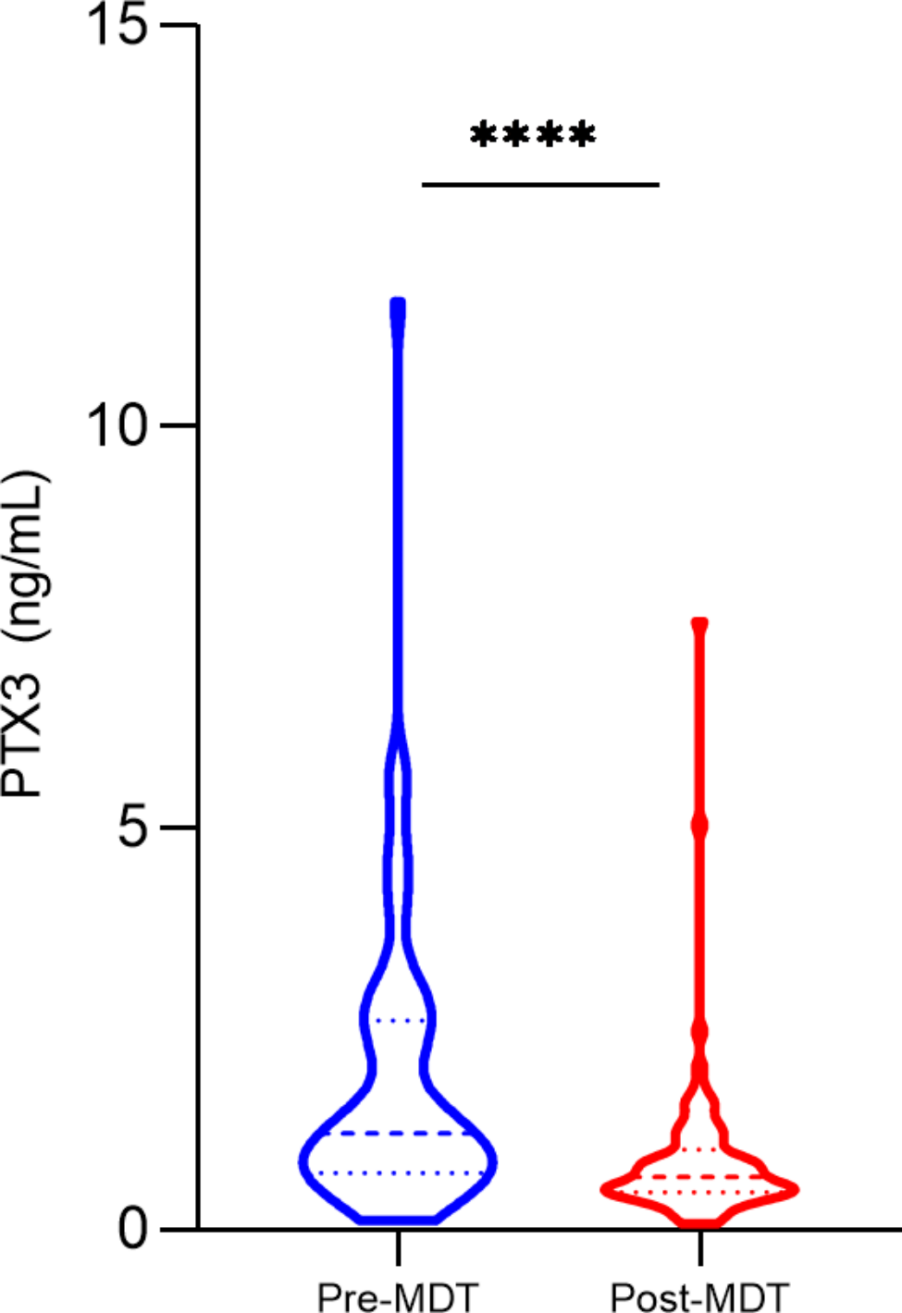
PTX3 plasma levels (ng/mL) in patients with leprosy before multidrug therapy (MDT) and post-MDT. Statistical significance was determined using the Mann-Whitney Test. ****P < 0.0001

For the measurement of plasma levels of PTX3, 142 leprosy patients, 52 household contacts, and 84 blood donors were included. In the comparison between the groups studied, the treatment-naive leprosy patients (n = 64) had higher PTX3 levels (in ng/mL) when compared to household contacts and blood donors (median, patients: 1.2 ng/mL, contacts: 0.8 ng/mL, blood donors: 0.9 ng/mL; leprosy versus contacts, *p* = 0.003; leprosy versus blood donors, *p* = 0.04) (Fig. 1). Plasma levels of PTX3 were also higher in pre-multidrug therapy (MDT) patients than post-MDT subjects (1.8 ng/mL versus 0.8 ng/mL, *p* < 0.0001) (Fig. 2).

**Fig. 3:**
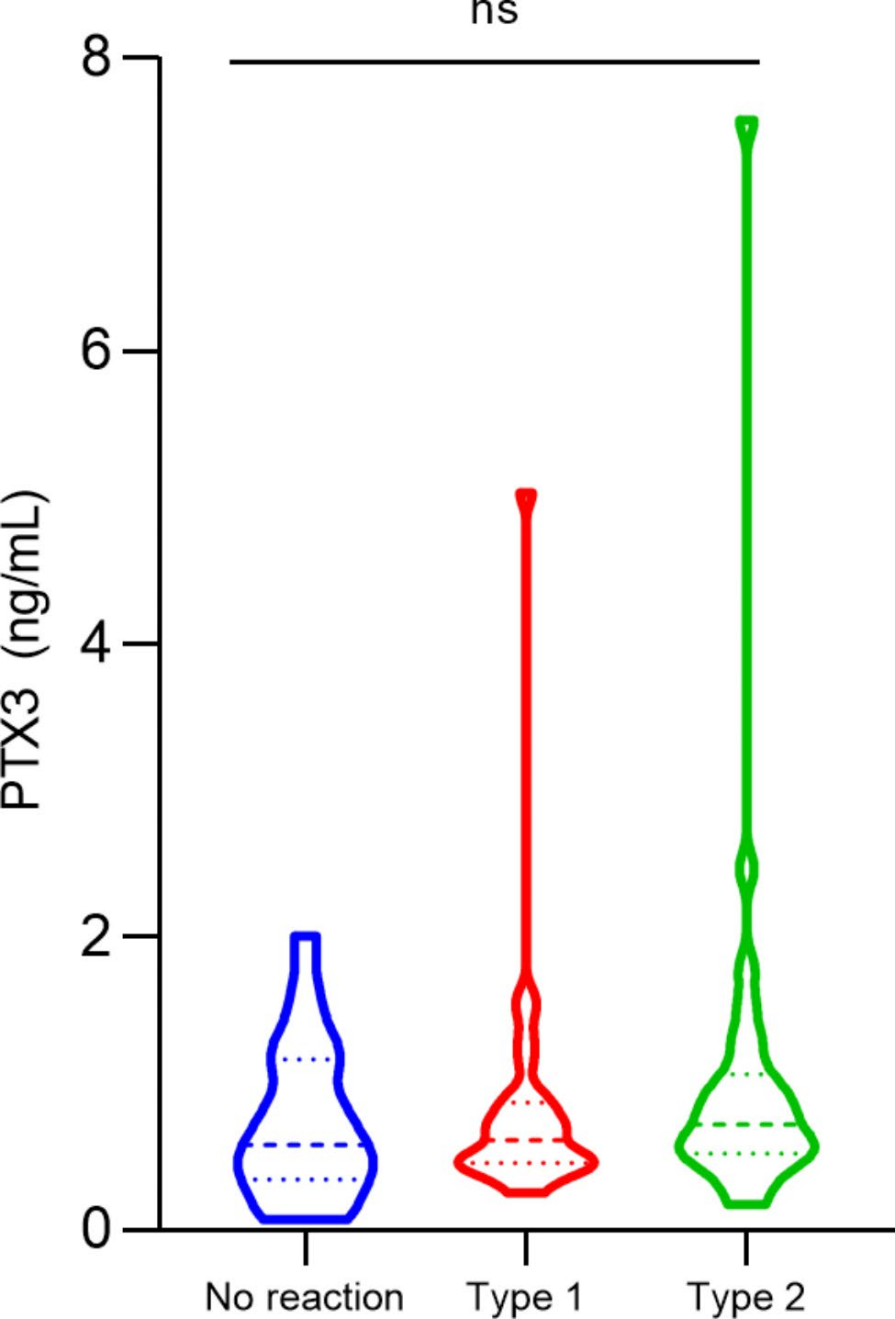
PTX3 plasma levels (ng/mL) distribution according to leprosy reactions. Statistical significance was determined using the Kruskal-Wallis test. ns: non-significant

**Fig. 4:**
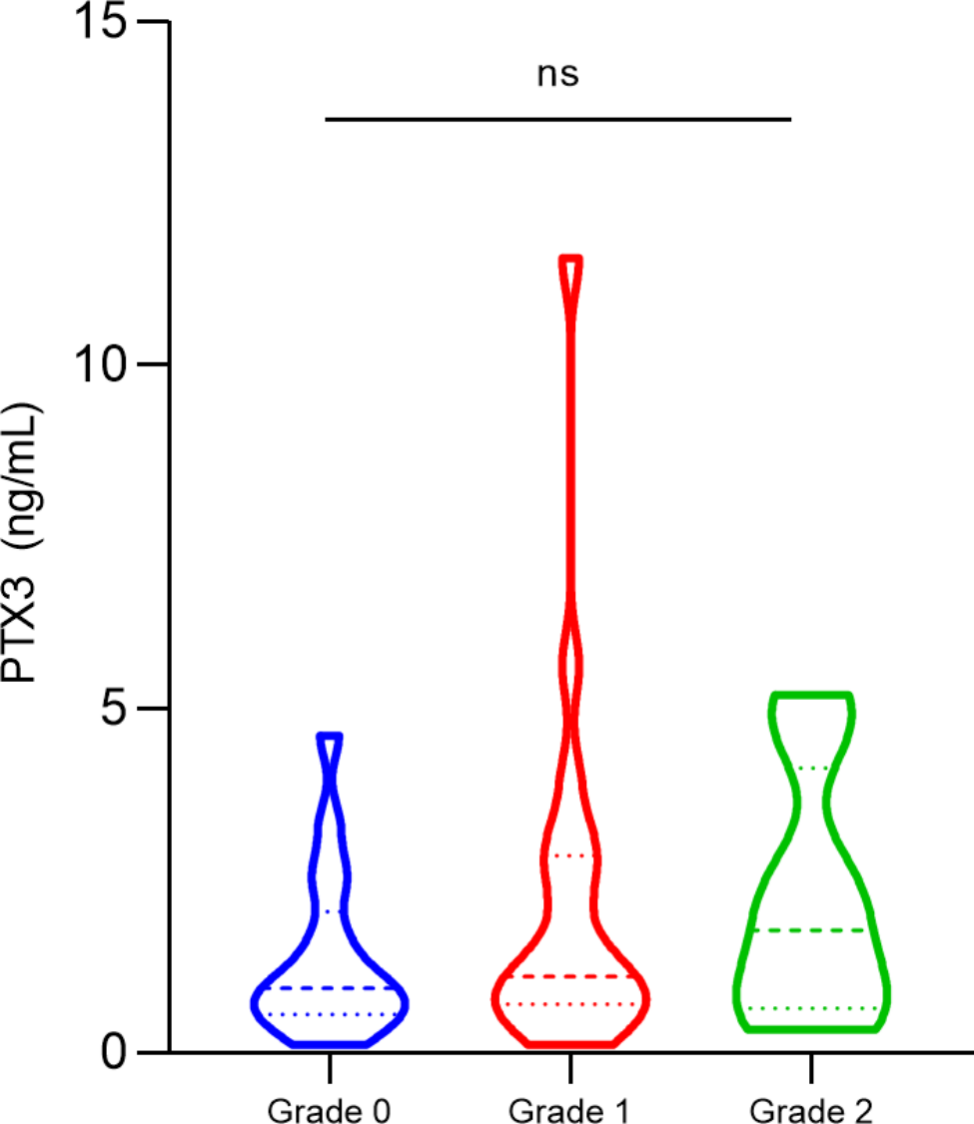
PTX3 plasma levels (ng/mL) distribution according to disability grade. Statistical significance was determined using the Kruskal-Wallis test. ns: non-significant

To assess the relationship of PTX3 levels with the occurrence of leprosy reaction, we included post-MDT leprosy patients who did not develop reaction or who developed type 1 or type 2 reactions. It was observed that patients with type 2 reaction had the highest levels of PTX3 (median, no reaction: 0.5 ng/mL, type 1: 0.6 ng/mL, type 2: 0.7 ng/mL, *p* = ns) (Fig. 3). There was no significant difference between PTX3 levels according to the degree of disability (*p* = ns) (Fig. 4).

## Discussion

In the present study, we demonstrated that individuals with leprosy have higher levels of PTX3 than healthy individuals, including household contacts without clinical manifestations or blood donors. In addition, PTX3 levels were found to be higher before MDT initiation.

Immunological and genetic factors of the host may influence susceptibility in leprosy, determining the disease risk [17]. Mannose-binding lectin (MBL), with its polymorphisms in *MBL2*, is an example of an acute-phase protein involved with the activation of the complement system by the lectin pathway, which has been extensively investigated in leprosy [18]. MBL appears to play a dual effect, where it may be associated with protection against infection, but may also have a negative effect facilitating bacillary proliferation and increasing the risk of multibacillary leprosy [18]. Therefore, considering that PTX3 is an important acute phase protein involved in the regulation of the complement system, it is likely that PTX3 may play an important role in modulating the immune response in leprosy.

*PTX3* expression is induced by inflammatory cytokines in various cell types, including endothelial cells and monocytes, increasing its levels early in the inflammatory process, preceding an increase in CRP [19]. In line with our findings, Azzurri et al. (2005) observed that patients with untreated tuberculosis had higher plasma levels of PTX3 than household contacts and community controls, and PTX3 levels dropped significantly after initiation of treatment [20]. In addition, PTX3 plasma levels were significantly higher in Egyptian patients with pulmonary tuberculosis than healthy individuals. Moreover, a significant positive correlation was found between PTX3 levels and degree of lung involvement [21]. Finally, Mendes et al. (2017) demonstrated that PTX3 serum levels of patients with multibacillary leprosy were approximately 3.2-fold higher than those among endemic controls, corroborating our findings [9]. These results suggest that plasma levels of PTX3 may be a potential biomarker of infection in individuals with suspected leprosy, such as those with skin lesions or sensory loss living in endemic areas. However, since PTX3 may be elevated in other infectious processes, the result would need to be interpreted with caution, considering other clinical aspects.

Previous studies have shown the association of SNPs in *PTX3*, mainly rs1840680 and rs2305619, with susceptibility to infectious diseases such as *Pseudomonas aeruginosa* in patients with cystic fibrosis [11], *Aspergillus fumigatus* in patients undergoing hematopoietic stem cell transplantation [12], fungal infections in solid organ transplant patients [13], urinary tract infections [14], and pulmonary infection by *Mycobacterium tuberculosis* [10, 21]. Other studies have demonstrated the involvement of *PTX3* in the immunopathology of some diseases, such as severe forms of COVID-19 [15] and chronic hepatitis C [22]. In the present study, we did not find differences in the frequencies of rs1840680 and rs2305619 polymorphisms between the groups analyzed or with clinical characteristics such as reaction occurrence or physical disabilities. It is possible that the small sample size, in the healthy controls group or in the clinical subgroups, may have contributed to the lack of association observed. Further studies in larger populations are needed to confirm these findings.

In this study, we did not observe any significant difference between PTX3 levels and the occurrence of leprosy reaction. However, our findings indicate that individuals with type 2 reaction had higher levels of PTX3 when compared to those with type 1 reaction or without reaction. These results corroborate the findings of the study by Mendes et al. (2017) [9], who demonstrated that individuals with type 2 reaction had increased levels of PTX3 before the development of the reaction, and that the levels decreased after treatment with thalidomide. In addition, they found that PTX3 serum levels were approximately 1.8 and 2.5 times higher in patients with type 2 reactions than in those with type 1 reactions and multibacillary or paucibacillary leprosy, respectively. In the present study, we demonstrated for the first time that treatment with MDT significantly reduced plasma levels of PTX3. Therefore, it is likely that PTX3 levels are underestimated in patients with the reaction, contributing to the lack of association observed here compared to the previous study in which patients were enrolled before MDT.

Our study has limitations, including the small sample size of household contacts and the absence of samples from patients with leprosy reaction before the start of MDT. On the other hand, this is the first study in the literature to determine the frequency of *PTX3* polymorphisms in leprosy patients and to demonstrate the influence of MDT on PTX3 levels.

## Conclusions

In summary, our results demonstrated that PTX3 levels are elevated in leprosy patients when compared to healthy household contacts or blood donors, and that PTX3 levels decrease after initiation of MDT. These findings confirm previous evidence that PTX3 may play an important role in the pathogenesis of leprosy. Additionally, this study contributes valuable support for the use of PTX3 as a diagnostic marker in suspected cases, akin to the way CRP is employed in clinical practice for infectious diseases. To fully explore its potential as a reliable biomarker of infection or as a predictor of type 2 response in leprosy, further comprehensive studies involving larger populations are imperative.

### Electronic supplementary material

Below is the link to the electronic supplementary material.

**Supplementary Material 1: Figure S1**. Association of PTX3 plasma levels (ng/mL) and PTX3 polymorphisms (A) rs1840680 and (B) rs2305619. Statistical significance was determined using the Kruskal-Wallis test. ns: non-significant

## Data Availability

The datasets used and/or analysed during the current study are available from the corresponding author on reasonable request.

## Abbreviations

CR: Complement receptor
CRP: C–reactive protein
ELISA: Enzyme–linked immunosorbent assay
HIV: Human Immunodeficiency Virus
HTLV: Human T–Cell Lymphotropic Virus
IL: Inteleukin
MB: Multibacillary
MBL: Mannose Binding Lectin
MDT: Multidrug therapy
PB: Paucibacillary
PCR: Polymerase Chain Reaction
PTX3: Pentraxin 3
SNP: Single Nucleotide Polymorphism
TLR: Toll–like receptor
TNF-α: Tumor Necrosis Factor-α
