# Supplementary material for: Association of PTX3 gene polymorphisms and PTX3 plasma levels with leprosy susceptibility
Source: BMC Infect Dis. 2023 Dec 5;23:853. doi: 10.1186/s12879-023-08862-0 (PMC10699025; doi:10.1186/s12879-023-08862-0)
Supplement: Supplementary file 1 — Supplementary Material 1: Figure S1. Association of PTX3 plasma levels (ng/mL) and PTX3 polymorphisms (A) rs1840680 and (B) rs2305619. Statistical significance was determined using the Kruskal-Wallis test. ns: non-significant [file 12879_2023_8862_MOESM1_ESM.docx]

**Supplementary Material**


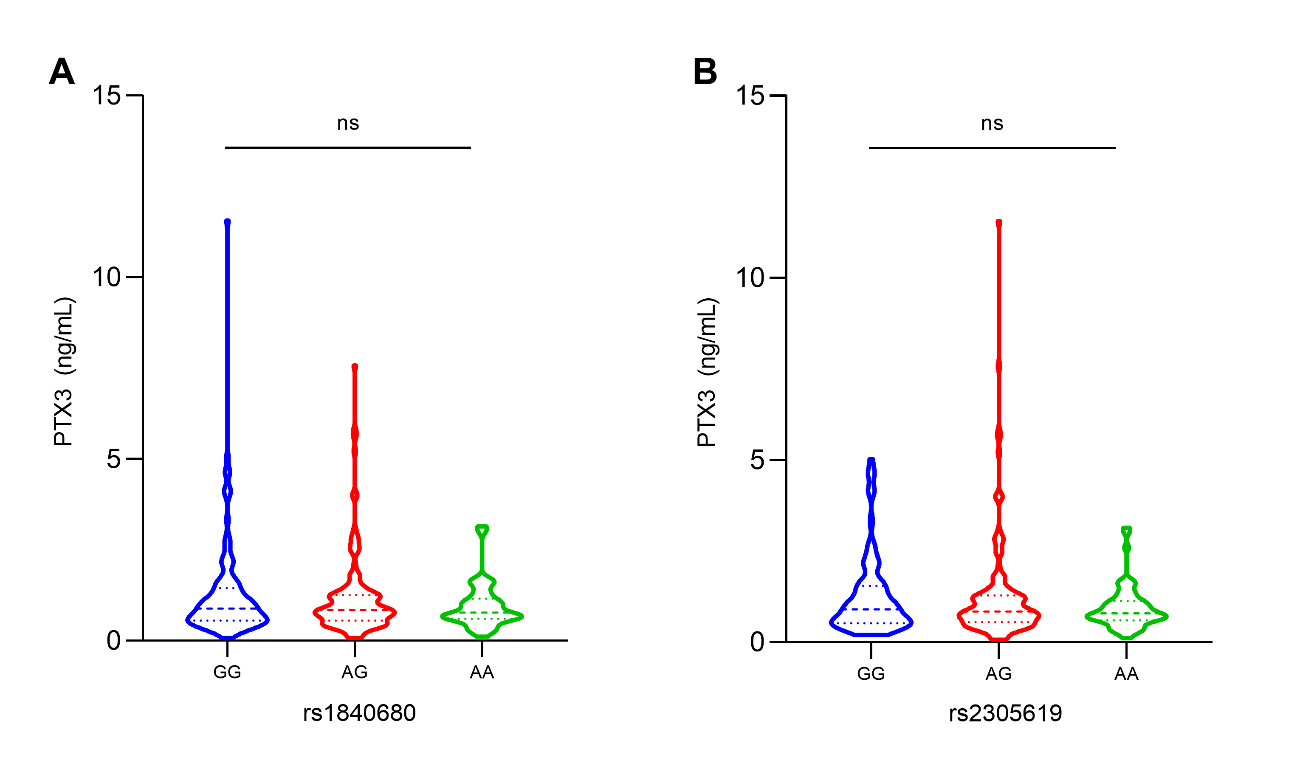
 **Figure S1.** Association of PTX3 plasma levels (ng/mL) and PTX3 polymorphisms (A) rs1840680 and (B) rs2305619. Statistical significance was determined using the Kruskal-Wallis test. ns: non-significant.
